# Twentieth Meeting of the Northern Ohio Dental Association

**Published:** 1879-07

**Authors:** L. C. Kelsey


### Proceedings of Societies.

### TWENTIETH MEETING OF THE NORTHERN OHIO DENTAL ASSOCIATION.

HELD IN CLEVELAND, OHIO, MAY 13 AND 14, 1879.

The meeting was called to order, at ten o'clock a. m., May 13th, by President J. W. Lyder, of Akron.

The minutes of the last annual meeting were read by the Secretary and approved.

The Treasurer and Secretary then made their reports, which were adopted.

Two vacancies in the Board of Examiners were filled by the appointment of Drs. L. Buffett and D. R. Jennings by the President.

A Committee on Ethics was appointed, consisting of Drs. J. E. Robinson and D. R. Jennings, to whom was referred the case of Dr. C. B. Knowlton, for unprofessional conduct in extensively advertising to do work at greatly reduced prices.

Dr. Jennings then read an interesting communication from Prof. J. Taft, of Cincinnati, which, on motion, was received and the Corresponding Secretary instructed to communicate with the Professor in relation to the same.

The Executive Committee then presented the following subjects for discussion:

First—The "New Departure."

Second—Mechanical Dentistry.

Third—Prevention of Decay in Teeth.

Fourth—Protracted Operations: Their effects upon the nervous system.

Dr. L. Buffett opened the discussion on the new departure by some very pertinent remarks. He said: The theory of Dr. Chase, that gold was the poorest material for filling teeth was, to his mind, an error. It is very true that in some cases gold is not the best filling material. In calcified or chalky teeth other materials than gold would be indicated; the manipulation of gold in such teeth would tend to break them down. Great judgment must be exercised as to the proper filling to be used in different classes and conditions of teeth. Filling teeth with plastic fillings is yet in its infancy. As yet it is mainly experimental. No doubt the outcome will be an advanced step. The profession is working towards plastic fillings, and there may be such progress and success in this direction that in twenty years from this time they may be in general use; but with our present knowledge gold is the standard filling.

Dr. Jennings said that he could not indorse all Dr. Buffet had said. Gold is *the* filling *now* and will be. He had no sympathy with this new departure theory or with its advocates. Dr. Chase, one of the champions of this new theory, knows very well that gold is the best filling material, and I do not think he is thoroughly honest. I think he is working more in favor of himself than for the general good of the profession.

Dr. Butler said that he had no special interest in this new departure theory. The advocates of this theory once used gold and considered it the best filling material. Now, to turn round and advocate something else as strongly, looks a little as if there was a selfish object in view. Advocating inferior substances for filling teeth is all there is of this new departure. Why this theory has been left unnoticed is more than I can tell; and in regard to this matter I would say that it is not new; gold was not the first material used to prevent decay in teeth; it is not many years since gold became the most approved material for filling, in some form. The only explanation I could make as to why the theory is championed so strongly is that *if* the advocates of it could ever do good work by using gold that time has now passed. They have found it an easy way to make money by the use of plastic fillings. I would be glad to hail a substance that would do away with so much hard labor; but until that presents itself the present method is the best.

Dr. Siddall said he had a thorough orthodox training in dentistry. When he first went to Oberlin, twenty-two years ago, he one day met President Finney, who, after learning that he was a dentist, asked him if he used amalgam, to which he responded that he did not. The President then promptly replied, You *ought* to, it has been the *salvation* of some of my teeth. Dr. Siddall further said, I am not in favor of the new departure, but at the same time it is foolish for me or any one to say that all teeth should be filled with gold.

On motion the association adjourned until two o'clock, p. m.

AFTERNOON SESSION.

The association was called to order at two o'clock by the President. Minutes of the morning session were read and approved.

The Committee on Ethics made their report in regard to the case of C. B. Knowlton, which was laid upon the table for future discussion.

The Committee of Examiners reported the following as suitable persons to become members of the association: Drs. H. Barnes, L. P. Holbrook, G. H. Wilson, T. W. Ensign and H. F. Harvey. On motion they were unanimously elected to membership.

The report of the Committee on Ethics was then taken from the table, and after a spirited discussion in regard to the unprofessional conduct of Dr. C. B. Knowlton, the report was, on motion, laid over for one year, and that in the meantime he (Dr. Knowlton) would be considered suspended from membership in the association.

The first subject, new departure, still being up for consideration Dr. Butler remarked that no one material would meet all cases in the best manner. Gold, although considered the best, will not answer in all cases. Then it is that plastic fillings, tin and other materials, come in and serve a good purpose. He had known dentists who had not used amalgam in a number of years; but this he considered the exception to the general practice. In the matter of filling teeth no undeviating rules can be laid down. The best intelligence of the operator must decide when and what kind of fillings are proper to be used.

Dr. W. P. Horton said that the tendency in all professions was to speculative methods. The stringent times had something to do with this state of things, and the cheaper the method the more feasible it seemed. Ninteen out of twenty patients can not tell when a good operation is performed. The new depature men have taken advantage of this ignorance and of the hard times to advertise their cheap materials, and have sometimes charged more for their operations than a first class dentist would charge.

Dr. Slosson—I have been out of regular practice for several years and would like to know definitely what the new departure means.

In reply to this inquiry Dr. L. Buffett read an article from Dr. Chase, which was wholly opposed to gold as a filling material.

Dr. Ambler said he was satisfied there was nothing new in dentistry. Herodotus found the practice of dentistry in vogue four hundred and forty-five years before Christ. There is no doubt but that gold is the best material, in first or second class hands for filling teeth, and he did not care to receive instruction from the advocates of the new departure.

On motion this subject was passed and the second on the list, Mechanical Dentistry, was taken up.

Dr. J. E. Robinson read an interesting and instructive paper on the subject, in which he discussed, at some length, the different bases for artificial dentures, giving the preference to continuous gum work. He thought the time not far distant when operative and mechanical dentistry would be separated into two distinct branches. The paper contended for an honest effort to build up instead of tearing down this important branch in dentistry.

Dr. Butler spoke briefly on the subject, referring to the methods and appliances in use, and requested those who had anything new on the subject to bring it before the association.

Dr. B. F. Gibbons said that the cause of the deterioration in mechanical dentistry was due to the efforts of speculative practitioners, and, as a result, the profession is flooded with a number of incompetent dentists, thereby compelling good ones to do cheap work in order to obtain a livelihood, and necessitating inferior methods and appliances.

Dr. Corydon Palmer spoke strongly in opposition to the practice of extracting teeth for the purpose of inserting artificial ones. He hoped all would study to avoid this practice. The extraction of teeth changes the features, and often makes men assume the appearance of age and injures articulation.

On motion the association adjourned to meet at eight o'clock in the evening.

EVENING SESSION.

The association was called to order at eight o'clock by the President.

The reading of the minutes was dispensed with and reports of committes called, the Examining Committee presented the names of Drs. T. S. Seeley, F. E. Lyon and B. F. Gibbons. On motion they were duly elected to membership.

The subject of Mechanical Dentistry still being under consideration Dr. L. Buffett said that he thought good would come out of the discussion of the various methods proposed. It should be the earnest desire of every one entering the profession to better his calling in some way, instead of pursuing it simply to line his pockets with a few dollars. The man who had no other than pecuniary motives in view would be a failure professionally; a weight instead of a help and benefit to humanity. In regard to the younger members of the profession he counselled them not to be confined to either celluloid or rubber as bases for artificial dentures, but to make use of such materials as experience and best judgment would dictate for particular cases.

Dr. Jennings—It sometimes happens that plates fit too closely, thereby causing irritation, no matter what material is used.

Dr. Siddall spoke of the difficulty encountered by practitioners in keeping up with the demands of the profession in all its branches. One who has his own operating and mechanical work to attend to would scarcely get much time to spend in putting up gold jobs or continuous gum work.

Dr. Robinson said he thought continuous gum far the best in a majority of cases. Gold was good for plates but he did not like silver though he considered even that better than rubber.

Dr. L. Buffett—In regard to what Dr. Siddall said I would remark that almost any one could find a little time for mechanical dentistry. In putting up one gold plate you would make more money than by putting up five or six rubber or celluloid plates. We should always strive to do the very best thing for our patients.

Dr. Butler—I am aware that excessive work always tends to break down the best of constitutions, but any dentist could keep up a regular practice at the chair and yet find time to do one piece of artificial work in gold each week. Any one who could not make nice artificial work would never make a dentist. Gold work is unquestionably the best for partial sets.

Dr. Jennings—I think we should start out with the question, What is best for our patients? Continuous gum work *is no doubt* the best thing to be worn in the mouth. By it, artistic skill can be displayed, and it is always free from unpleasant odor. I can ditect rubber plates by the breath of the wearer. We need to cultivate and encourage a higher grade of artistic skill and beauty in mechanical dentistry.

Dr. Harvey said that at Ann Arbor, Michigan, various methods were followed, alluminium, with rubber attachment, being one of those taught in the dental department and he was well pleased with this method.

Dr. Gibbons—In our discussions of this subject one important matter has been overlooked. I refer to the articulation of sounds. Imperfection of speech is often due to improper dentures. The thicker the plate the more difficult would it be for the patient to speak with ease. I think Allen's continuous gum work the most perfect and less liable than any other to interfere with the articulation of sounds.

Dr. Holbrook thought continuous gum work was gaining favor among the people. Rubber plates often irritate and overheat the mouth, and when this occurs gold plates nicely struck up will remedy this condition.

Dr. Buffett said there was greater absorption under a rubber plate than under a metal plate and the closer a plate fitted the greater would be the absorption.

Drs. Seeley, Barnes and Tunison took part in the discussion of this subject, and expressed their preference for continuous gum work.

On motion the subject was passed and the association adjourned to meet at nine o'clock a. m., Wednesday, May 14th.

MORNING SESSION—WEDNESDAY, MAY 14.

Meeting called to order at nine o'clock by the President.

The third subject, Prevention of Decay in Teeth, was then taken up.

Dr. Jennings opened the discussion by saying that this subject is a very broad one, and has many phases. Different methods and theories are adopted in order to prevent decay. Cleanliness performs a very important part in the preservation of the teeth. Separating teeth is often beneficial, as too close contact of one tooth with another often causes decay. Where decay commences at the free margin of the gums, nitrate of silver may sometimes be used with good results, as it tends to neutralize lactic acid. Brushes and tooth picks may perform a very important part in the prevention of decay.

Dr. Butler said Dr. Watt has given as clear a chemical analysis of the causes of decay in the teeth as any one in the country, but with all our light and investigation, we still know but little about these causes. The important question is, how, with our present knowledge, shall we arrest decay? What shall we do and advise in order to secure the best results? We must insist upon cleanliness, the free use of the brush plays a very important part in keeping the teeth from decay. How to arrest the abrasion or the decay of teeth is a very important enquiry, for we can not always tell what elements or conditions are most injurious to tooth structure, whether they are of an acid or alkaline nature.

Dr. Ambler said he was not in favor of Dr. Arthur's method, which consisted in cutting away the decayed surface and then polishing to prevent further decay. When teeth were decayed we should resort to cohesive gold as a *standard* filling. Even soft and chalky teeth may be filled with it and made useful, but in such teeth free separations should be made so that they may be thoroughly cleaned. As a rule I prefer to fill soft teeth with tin foil. He then related several instances where he had filled teeth with tin, which he thought rendered them far more durable than if they had been filled with gold.

Dr. Holbrook—I often partially fill teeth with oxy-chloride of zinc, then complete with gold. Sometimes I use tin and gold combined as a filling.

Dr. Seely—In filling soft and chalky teeth I make use of Hill's Stopping. Am careful to put it in very dry; endeavor to see my patients often, and in a few months, the conditions being favorable I remove most of the stopping and fill with gold or tin.

Dr. Palmer—I have not much to say as to the causes of decay in the teeth, but will say something as to its prevention. I have no confidence in the new departure or in its advocates. With me cohesive gold stands at the head as a filling material. Tin foil stands next in value. Laterally there has been much improvement in amalgams but I seldom use of any of them. Fletcher's preparation of zinc I prefer to any other plastic filling.

Dr. L. Buffett—We need to look into the causes of decay. Hereditary taint has much to do in causing the decay of teeth. Lack of vitality in the system, defective physical organization plays an important part in the deterioration or decay of teeth. I believe there is great deterioration of teeth. The third molars are fast becoming mere rudimentary teeth, and how to counteract this tendency is a very important question. One important thing is to improve the health of the patient and thereby improve every tissue of the body. Our mission is not to destroy but to preserve. Destroy the nerve of a tooth and it soon softens, its integrity is gone. We should make use of every means to improve the general health.

On motion this subject was passed.

Dr. J. E. Robinson made a motion that when the association adjourns it shall be to hold its next annual meeting at Akron, Ohio, on the second Tuesday and Wednesday of May, 1880.

Carried.

The association then proceeded to the election of officers for the ensuing year which resulted as follows:

For President—J. W. Lyder. of Akron, Ohio.

For Vice-President—J. S. Chandler, of Sandusky, Ohio.

For Corresponding Secretary—H. F. Harvey, of Cleveland, Ohio.

For Recording Secretary—L. C. Kelsey, of Elyria, Ohio.

Treasurer—J. E. Robinson, of Cleveland, Ohio.

Dr. J. W. Lyder, the out-going and in-coming President, then made a few very appropriate remarks.

On motion the President was instructed to appoint three members of the association to designate delegates to attend the American Dental Association, whereupon the following were appointed: Drs. C. R. Butler, L. Buffett and A. Terry.

The President then appointed the following Committees:

On Board Examiners—Drs. D. R. Jennings, J. F. Siddall, and D. Gibbons.

On Executive Committee—Drs. L. Buffett and D. R. Jennings.

Committee on Ethics—Drs. J. E. Robinson, L. Buffett and D. R. Jennings.

Delegates elected to attend the American Dental Association as follows: Drs. H. F. Harvey, L. Buffett, J. W. Lyder, C. R. Butler, C. Palmer and H. E. Lyon.

On motion the Secretary, L. C. Kelsey, was allowed ten dollars for past services.

The association then adjourned, to meet at two o'clock, p. m.

LAST SESSION.

Meeting was called to order at two o'clock p. m, President Lyder in the Chair.

On motion Dr. L. Buffett was appointed a committee to solicit funds for the "McQuillan Testimonial." Most of the dentists present generously responded to the call.

Miscellaneous business was then called up. Several cases were reported and discussed in a free and informal manner, most of the members participating.

The last subject, Protracted Operations: Their effects upon the nervous system, was then taken up.

Dr. Butler opened the discussion by saying that he would like to know what range the subject was to take; whether it has especial reference to the patients or to the operator, or to both. Within a few years the instruments, appliances, etc., introduced have rendered difficult operations more easy than formerly. Even building up crowns with gold have now become a moderate operation. Yet, with all our helps, there is a strain upon operator and patient which is very exhausting. I seldom attempt to do a difficult operation at one sitting. At first I look the case over carefully; prepare the cavity, and complete the operation at another time. In large operations there is always a strain upon the operator by the close attention he gives to his work, and by the anxiety he feels for the comfort of his patient. There is also a drain upon the nervous force of the patient by the posture, tension of the mind, and the surroundings. We should guard against little annoyances to our patients.

Dr. C. Palmer said he was glad this subject was up for discussion. We need to understand what our patients can bear, and should be careful not to overtask their nervous force. I divide up my operations and scarcely ever work over two hours at a time on one patient. One thing we ought to understand, we must gain the confidence of our patients and be in mental harmony and sympathy with them; then it is that our work is made comparatively easy.

Dr. Jennings—I am satisfied we do not husband our resources enough. Few can long stand the strain of working day in and day out at the chair. We should make short engagements, favor our patients as much as possible, and show sympathy for them. Three hours is long enough to work over one patient at a time, and if the patient should show signs of nervous prostration, the time should be shortened.

Dr. Lyder—Frequently persons come to me for immediate work, but I do not always comply with their wishes in that respect. I like to diagnose my cases, understand their peculiarities, and get acquainted with them. As a general rule short and light operations with strange patients should be made.

Others participated in this discussion, and thus ended one of the most interesting and profitable meetings the association ever held.

On motion, the Corresponding Secretary was instructed to have printed a form of certificate for delegates to the American Dental Association.

The association then adjourned to hold its next annual meeting at Akron, Ohio, on the second Tuesday and Wednesday of May, 1880. L. C. KELSEY, Secretary.

### Editorial.

### AMERICAN DENTAL ASSOCIATION.

